# Does antiretroviral therapy use affect the accuracy of HIV rapid diagnostic assays? Experience from a demographic health and surveillance site in rural South Africa^☆^^☆☆^

**DOI:** 10.1016/j.diagmicrobio.2020.115031

**Published:** 2020-06

**Authors:** Mark J. Siedner, Kathy Baisley, Olivier Koole, Innocentia Mpofana, Gregory Ording-Jespersen, Philippa Matthews, Kobus Herbst, Theresa Smit, Deenan Pillay

**Affiliations:** a Africa Health Research Institute, KwaZulu-Natal, SA; b Massachusetts General Hospital, Boston, USA; c Harvard Medical School, Boston, USA; d University of Kwazulu-Natal, Durban, SA; e London School of Tropical Medicine and Hygiene, London, UK; f University College London, London, UK

## Abstract

Rapid diagnostic tests (RDTs) are the mainstay of HIV diagnosis in the developing world but might have poor sensitivity among individuals taking antiretroviral therapy (ART). We leveraged a home-based HIV testing program linked to clinical data to compare the sensitivity of RDTs between individuals using versus not using ART. Field workers tested 6802 individuals using 2 HIV RDTs, which were compared to a single HIV immunoassay tested on dried blood spots. Approximately 5% (371/6802) tested positive by immunoassay, of whom 157 (42%) were currently on ART. The sensitivity of the Abon RDT among those never versus currently on ART was 91.6% (95% CI 88.3–94.3) and 96.6% (95% CI 88.3–94.3), respectively, and 95.4% (95% CI 92.8–97.3) versus 99.3% (95% CI 95.2–99.7) for the Advanced Quality assay. We report similar sensitivity of RDTs in ART-naïve and ART-experienced individuals, which mitigates concerns about their use among treated individuals in population-based epidemiologic surveys and those transferring care.

## 1 Background

The World Health Organization supports use of rapid diagnostic tests (RDTs) for diagnosis of HIV in settings where laboratory-based confirmatory assays are not available (Consolidated guidelines on HIV testing services, 2015). RDTs allow rapid, low-cost, point-of-care diagnostic evaluation for HIV without need for complex laboratory infrastructure or extensive human resource expertise. These characteristics make them a cornerstone of HIV diagnostic in much of the developing world. Recently updated WHO guidelines in 2019 now suggest use of 3 sequential positive tests that rely on excellent sensitivity and moderately high specificity of RDTs (World Health Organization, 1997; 2019).

RDTs have been primarily developed and evaluated to make new diagnoses of HIV. More recently, such assays have been applied at a population scale within generalized epidemics, for the purpose of HIV surveillance (Kim et al., 2016) among individuals transferring care who sometimes do not disclose their ART status (Grabowski et al., 2018; Manne-Goehler et al., 2019; Sykes et al., 2019), to determine inclusion criteria for research studies (Coleman et al., 2018) and in the context of community-based test-and-treat ART initiatives (Hayes et al., 2019). These scenarios will increasingly include individuals on ART. However, the high sensitivity of RDTs for the detection of HIV among people on ART has been challenged. Although a relatively rare phenomenon in practice, early initiation of ART during acute HIV infection prevents development of an antibody response to HIV (de Souza et al., 2016). Some studies have also suggested antiretroviral therapy use might decrease the sensitivity of these assays (O'Connell et al., 2003; Merchant et al., 2014; Fogel et al., 2017). The biological mechanism for declining sensitivity of RDTs is that the titers of anti-HIV envelope and other antigens decline after years of ART use (Merchant et al., 2014; Fogel et al., 2017). If true, high rates of false-negative RDT results would have important implications both for population-based epidemiologic studies and for testing patients currently or previously in care, which is commonly done at the time of clinic transfer. Repeat testing of individuals who have surreptitiously transferred care or are seeking to enrol in studies as ART naïve is widely reported in both clinical and programmatic settings (Fogel et al., 2013; Sullivan et al., 2013; Coleman et al., 2018).

We sought to answer 2 questions: 1) What proportion of individuals actively taking ART test positive by RDT and HIV 1/2 antigen/antibody enzyme immunoassay? 2) Is the sensitivity of RDTs, compared to HIV 1/2 antigen/antibody enzyme immunoassays, decreased among individuals taking ART versus those ART naïve? To do so, we leveraged a demographic health and surveillance (DHS) program that routinely performs home-based HIV testing using RDTs with paired HIV 1/2antigen/antibody immunoassays on dried blood spot (DBS) specimens. We hypothesized that, in a programmatic setting in rural South Africa, home-based RDTs would perform equally well among those currently taking or naïve to ART.

## 2 Methods

### 2.1 Study design

The African Health Research Institute (AHRI) (formerly the Africa Centre for Health and Population Studies) is a Wellcome Trust–funded research institute in South Africa. In 2000, AHRI established a DHS in rural uMkhanyakude District, northern KwaZulu-Natal, which now covers an area of 845 km^2^ with a population of approximately 150,000 (Tanser et al., 2008). Annual household-based surveys are used to collect information on births, deaths, and migration patterns for all household members, including nonresidents. Resident members who are aged ≥15 years are also invited to participate in an individual survey, which includes an interview on general health and sexual behavior, and collection of a DBS for anonymized HIV testing. In addition, HIV counseling and testing (HCT) using RDTs is offered to all residents aged ≥15 years. Although we encourage testing in individuals who do not know their status, we do not exclude testing among individuals who have a recent negative test or have had prior positive test result. Participants who newly test HIV positive are referred for care at 1 of the 11 government primary health care clinics in the surveillance area.

AHRI has a memorandum of agreement with the KwaZulu-Natal Provincial and District Department of Health to receive data from an HIV care electronic patient record system that is used in the government clinics (TIER.net). The TIER.net database contains information on clinic visit attendance, laboratory results, and ART dispensing records for all patients on ART. Clinical records of patients in the TIER system are linked with their household and individual-level data gathered through the AHRI demographic surveillance system. Individuals are linked using their unique South African identification number or by first name, surname, age, and sex using algorithms developed by AHRI. On a monthly basis, TIER.net data are transferred to AHRI from the central Department of Health. Individuals who had any record of receiving ART in TIER were considered ever on ART. Those who had initiated ART at least 1 year before the RDT and who had attended a clinic for ART care in the last 6 months were considered to be currently on ART.

### 2.2 HIV testing methods

Participants in the DHS program who consent to home-based HCT undergo a finger-prick (capillary) blood sample collected by a trained lay field worker for testing with 2 RDTs in parallel: the Abon™ HIV 1/2/O Tri-Line (Abon Biopharm, China) and Advanced Quality™ Rapid Anti-HIV (1&2) Test (InTec, China). All RDTs are performed during the household visit by trained counselors, and pre- and posttest counseling is provided. For individuals with discordant RDT results, a venous blood sample is collected for a third lab-based ELISA immunoassay (EIA) test to determine a clinical result. All individuals also receive an additional confirmatory test at the time of presentation to clinic for initiation to care. The use of parallel testing differs from WHO and provincial guidelines and was instituted to reduce the risk of false-positive testing. All participants who consent to the HIV surveillance also provide a capillary blood specimen for DBS preparation, which is tested using a fourth-generation EIA (Genscreen ULTRA HIV Ag-Ab enzyme immunoassay; Biorad, Marnes-la-Coquette, France). All EIA tests are performed at the AHRI Diagnostic Laboratory in Durban, South Africa.

Although the Genscreen EIA is not validated by the manufacturer for use on DBS samples, the assay has been validated in-house in our laboratory. Briefly, a 4.7-mm DBS punched spot is eluted overnight (max 16 h) at 4 °C in 200 μL of PBS (with no additives). A 50-μL aliquot of the eluent is used as the sample. The assay is performed as per the manufacturer’s protocol for plasma and serum and run against the controls provided in the assay. The threshold used for a positive result for testing of DBS samples has been adjusted to twice the standard assay threshold.

### 2.3 Statistical analysis

We categorized the cohort into those never, currently, or previously on ART and summarized demographic and clinical indicators. For this analysis, we calculated the sensitivity and specificity of both RDTs compared to the EIA immunoassay performed on DBS samples as a reference standard for the total cohort, and stratified by ART use categories. We compared the sensitivity of each assay in individuals currently on ART to those individuals never on ART using a Fisher’s exact test.

In a secondary analysis, true positive (reference) was defined by either a positive test on our EIA or a record of ART use in the TIER electronic medical record, whereas a true negative was defined as negative immunoassay and no record of ART care. Sensitivity and specificity of each RDT, and of the parallel strategy, were calculated among all individuals and stratified by ART status, as described above.

### 2.4 Ethics

Ethical approval for the demographic surveillance study, linkage to the government ART records (TIER.Net), and analyses of these data was granted by the Biomedical Research Ethics Committee of the University of KwaZulu-Natal, South Africa. Separate informed consent was obtained for the main household survey, the individual-level questionnaires, HCT, and provision of the DBS.

## 3 Results

The study population comprised 6802 individuals who accepted HCT and provided a DBS specimen between 1 June and 20 December 2017; median age was 35 years (Table 1). The majority (4778, 70%) were female, and most (6613, 97%) had never been on ART, as determined by the TIER.Net electronic record. Among the 189 who had ever been on ART, 157 (83%) were currently on ART for a median duration of 4.9 years. There was no evidence of a difference in age between individuals on ART and those who had never been on ART (*P*=0.19, by Wilcoxon rank sum test), but a higher proportion of those on ART were female (90% versus 70%, respectively, *P*<0.001 by *χ*^2^ test).

**Table 1:** Cohort characteristics.

	All participants *n*=6802	Participants never on ART^a^ *n*=6613	Participants with any current or prior ART^b^ *n*=189	Participants on ART >12 months^c^ *n*=157
**Age (years)**				
<25	2303 (33.9%)	2279 (34.5%)	24 (12.7%)	18 (11.5%)
25–34	1080 (15.9%)	1019 (15.4%)	61 (32.3%)	47 (29.9%)
35–44	588 (8.6 %)	552 (8.3 %)	36 (19.0%)	32 (20.4%)
45–54	732 (10.8%)	697 (10.5%)	35 (18.5%)	33 (21.0%)
55+	2099 (30.9%)	2066 (31.2%)	33 (17.5%)	27 (17.2%)
Median (IQR)	35 (21–58)	35 (21–59)	36 (28–49)	38 (29–50)
**Sex**				
Male	2024 (29.8%)	2001 (30.3%)	23 (12.2%)	16 (10.2%)
Female	4778 (70.2%)	4612 (69.7%)	166 (87.8%)	141 (89.8%)
**Residence**				
Urban	224 (4.9 %)	216 (4.9 %)	8 (7.2 %)	7 (6.9 %)
Periurban	1250 (27.5%)	1218 (27.5%)	32 (28.8%)	31 (30.7%)
Rural	3071 (67.6%)	3000 (67.7%)	71 (64.0%)	63 (62.4%)
*Missing*	*2257*	*2179*	*78*	*56*
**Marital status**				
Single	862 (14.2%)	849 (14.5%)	13 (7.1 %)	12 (7.8 %)
Married/informal union	4330 (71.5%)	4181 (71.2%)	149 (81.4%)	122 (79.7%)
Widowed/separated/divorced	865 (14.3%)	844 (14.4%)	21 (11.5%)	19 (12.4%)
*Missing*	*745*	*739*	*6*	*4*
**Education**				
None	1605 (34.1%)	1577 (34.4%)	28 (23.5%)	23 (21.7%)
Less than complete secondary	1692 (35.9%)	1651 (36.0%)	41 (34.5%)	36 (34.0%)
Complete secondary/above	1410 (30.0%)	1360 (29.6%)	50 (42.0%)	47 (44.3%)
*Missing*	*2095*	*2025*	*70*	*51*
**Employed**				
No	3277 (85.7%)	3182 (85.8%)	95 (84.1%)	86 (84.3%)
Yes	545 (14.3%)	527 (14.2%)	18 (15.9%)	16 (15.7%)
*Missing*	*2980*	*2904*	*76*	*55*
**Household SES tertile**				
Low	1453 (31.8%)	1415 (31.8%)	38 (33.9%)	35 (34.3%)
Middle	1629 (35.7%)	1587 (35.6%)	42 (37.5%)	38 (37.3%)
High	1484 (32.5%)	1452 (32.6%)	32 (28.6%)	29 (28.4%)
*Missing*	*2236*	*2159*	*77*	*55*
**Time on ART (years)**				
Median (IQR)	-	-	4.6 (2.4–6.9)	4.9 (2.6–7.0)
**CD4 at first clinic visit**^d^				
Median (IQR)	-	-	340 (171–464)	326 (167–445)
*Missing*			*12*	*7*
**CD4 at ART initiation**				
Median (IQR)	-	-	245 (131–424)	221 (130–410)
*Missing*			*43*	*28*
**CD4 at last clinic visit**^d^				
Median (IQR)	-	-	500 (367–712)	497 (367–727)
*Missing*			*12*	*7*

a Individuals who have no record in TIER.

b Individuals who have a record in TIER of ever being on ART.

c Individuals who have a record in TIER of an ART visit <6 months before the rapid test and have been on ART for >1 year.

d First/last recorded CD4 count at clinic visits for HIV care

**Fig. 1:**
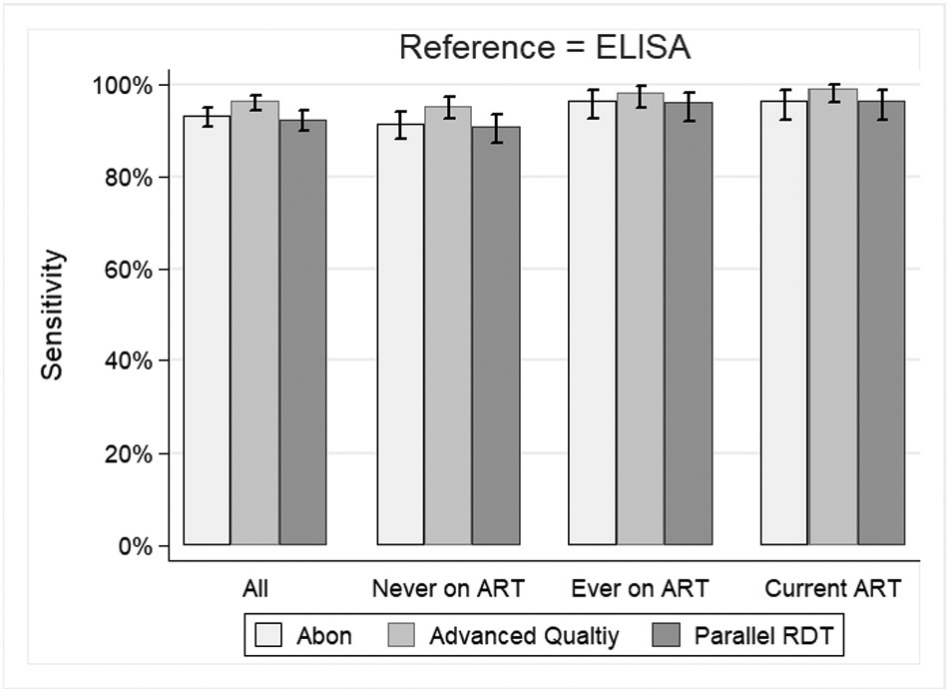
Sensitivity (95% CI) of rapid diagnostic tests by ART status.

**Table 2:** Sensitivity and specificity of rapid diagnostic tests.

	Reference = ELISA^a^				Reference = ELISA + TIER^b^			
Assay	Test + / True +	Sensitivity	Test − / True −	Specificity	Test + / True +	Sensitivity	Test − / True −	Specificity
**All participants**								
Abon RDT	513 / 550	93.3 (90.8–95.2)	6238 / 6246	99.9 (99.7–99.9)	513 / 560	91.6 (89.0–93.8)	6228 / 6236	99.9 (99.7–99.9)
Advanced Quality RDT	530 / 550	96.4 (94.4–97.8)	6245 / 6250	99.9 (99.8–100.0)	530 / 560	94.6 (92.4–96.4)	6235 / 6240	99.9 (99.8–100.0)
Parallel RDT^c^	509 / 550	92.5 (90.0–94.6)	6240 / 6244	99.9 (99.8–100.0)	509 / 560	90.9 (88.2–93.1)	6230 / 6234	99.9 (99.8–100.0)

a ELISA testing of DBS sample used as reference (‘true’ status).

b Reference is based on ELISA results and presence of record in TIER. Individuals with positive ELISA or record in TIER are classified as HIV positive; individuals with negative ELISA and no record in TIER are classified as HIV negative.

c Test positive defined as being positive on both rapid tests (Abon and Advanced Quality); test negative defined as being negative on at least one of the rapid tests.

**Table 3:** Sensitivity of rapid diagnostic tests by ART status.

	Reference = ELISA^a^			Reference = ELISA + TIER^b^		
Assay	Test + / True +	Sensitivity	*P* value^c^	Test + / True +	Sensitivity	*P* value^c^
**Never on ART**^d^						
Abon RDT	340 / 371	91.6 (88.3–94.3)	-	340 / 371	91.6 (88.3–94.3)	-
Advanced Quality RDT	354 / 371	95.4 (92.8–97.3)	-	354 / 371	95.4 (92.8–97.3)	-
Parallel RDT^e^	337 / 371	90.8 (87.4–93.6)	-	337 / 371	90.8 (87.4–93.6)	-
**Any current or prior ART**^f^						
Abon RDT	173 / 179	96.6 (92.8–98.8)	0.03	173 / 189	91.5 (86.6–95.1)	>0.99
Advanced Quality RDT	176 / 179	98.3 (95.2–99.7)	0.14	176 / 189	93.1 (88.5–96.3)	0.32
Parallel RDT^e^	172 / 179	96.1 (92.1–98.4)	0.04	172 / 189	91.0 (86.0–94.7)	>0.99
**Participants on ART >12 months**^g^						
Abon RDT	143 / 148	96.6 (92.3–98.9)	0.05	143 / 157	91.1 (85.5–95.0)	0.87
Advanced Quality RDT	147 / 148	99.3 (96.3–100.0)	0.03	147 / 157	93.6 (88.6–96.9)	0.39
Parallel RDT^e^	143 / 148	96.6 (92.3–98.9)	0.03	143 / 157	91.1 (85.5–95.0)	>0.99

a ELISA testing of DBS sample used as reference (‘true’ status).

b Reference is based on ELISA results and presence of record in TIER. Individuals with positive ELISA or record in TIER are classified as HIV positive; individuals with negative ELISA and no record in TIER are classified as HIV negative.

c *P* value comparing with group never on ART using Fisher’s exact test.

d Individuals who have no record in TIER.

e Test positive defined as being positive on both rapid tests (Abon and Advanced Quality); test negative defined as being negative on at least 1 of the rapid tests.

f Individuals who have a record in TIER of ever being on ART in TIER (includes individuals who are currently on ART).

g Individuals who have a record in TIER of an ART visit <6 months before the rapid test and have been on ART for >1 year.

All 6802 individuals completed EIA testing, of whom 6252 (91.9%) tested negative and the remaining 550 (8.1%) tested positive (Supplementary Table 1). Of these, 6796 (99.9%), 6800 (>99.9%), and 6794 (99.9%) also completed testing by Abon, Advanced Quality, and both tests in parallel, respectively. The sensitivity of the Abon assay among individuals currently on ART was 96.6% (95% CI=92.3–98.9%) and 91.6% (95% CI=88.3–94.3%) among those never on ART (*P*=0.05) (Table 2 and Fig. 1). Similarly, sensitivity of the Advanced Quality assay was higher among those on ART versus those never on ART (99.3%; 95% CI=96.3–100.0% and 95.4%; 95% CI=92.8–97.3%, respectively) (*P*=0.03). When both immunoassay and ART records were used as the reference standard, the sensitivity of the individual assays and parallel RDT strategy decreased slightly (Table 3), but there was no evidence of a difference in the sensitivity of any of the assays in individuals on ART compared with those never on ART.

## 4 Discussion

Home-based HIV RDT assays conducted by field workers in rural South Africa had a sensitivity among people taking ART for a median of 5 years that is equal to or better to that when used among ART-naïve individuals. We found that approximately 95% of HIV enzyme immunoassay-positive individuals actively taking ART in the public sector in South Africa tested positive by RDT compared to approximately 92% in those without a history of ART use. While we identified an overall imperfect sensitivity of the RDT assays, which requires additional attention, our results do not suggest that long-term ART has a major impact on the sensitivity of RDTs in this setting.

The first study to suggest a lower sensitivity of RDTs for detection of HIV infection among those on ART found a sensitivity of only 89% among those on ART (*n*=91) (O'Connell et al., 2003) compared to 100% sensitivity among those not on treatment (*n*=10, Table 4). Notably, the study assessed a second-generation assay that solely used recombinant gp41 envelope proteins as the test antigen, and the 4 false-positive specimens had low titers of anti gp41 antibodies in retrospective testing. The lowest sensitivity of RDTs among those on ART is also reported in a study of children and adolescents (*n*=27) who underwent repeated testing over calendar time, and found a false-negative testing rate ranging from 2% to 20%, depending on the assay (Merchant et al., 2014). This study also reported decreasing titers by enzyme immunoassay with increased duration of ART use, which was up to 15 years in duration, in many participants. Finally, a substudy of the HPTN 051 clinical trial assessed the validity of third-generation RDTs with multiple antigens among 207 adults with prospectively collected specimens (Fogel et al., 2017). Ten of 207 specimens (5%) were either nonreactive (*n*=1) or weakly positive by RDT (*n*=9) at follow-up testing, for a sensitivity of 99.5 and 95.1%, respectively, depending on whether or not weakly positive bands were considered positive. Four of the 10 individuals also had indeterminate or negative Western blot results, suggesting a more extensive effect of ART on production of anti-HIV antibodies. Counterintuitively, the frequency of weak or nonreactive bands was lower among those in the early ART arm (350–550 cells/μL) versus the delayed ART arm (CD4 <250 cells/μL) with 7/180 (3.9%) and 3/ 27 (11.1%, *P*=0.13), respectively. In our study which observed individuals in routine care, most individuals started ART with a low CD4 count (median 234), including those who had false-negative RDT results (median 109). Importantly, neither of the 2 latter studies included a control group of individuals who were also retested without exposure to ART, so whether the decrease in accuracy was due to ART or variations is test performance with repeat testing cannot be determined.

**Table 4:** Summary of studies that assessed the sensitivity of HIV rapid diagnostic tests (RDTs) among individuals on antiretroviral therapy (ART).

Author, year (REF)	Assay(s) tested	Analyte tested	Participants on ART	Sensitivity of RDTs among those on ART (95% CI)	Participants not on ART	Sensitivity of RDTs among those not on ART (95% CI)
O'Connell et al., 2003	OraQuick	Serum	91	**95.6% (89.1–98.8%**	10	100% (69.1–100%)
O'Connell et al., 2006	Multispot HIV-1/HIV-2	Serum	248	**100% (98.5–100%)**	0	n/a
Piwowar-Manning et al., 2014	OraQuick	Not specified	101	**98.0% (93.0–99.8%)**	0	n/a
	Uni-Gold	Not specified	101	**98.0% (93.0–99.8%)**	0	n/a
	Recombigen	Not specified	101	**98.0% (93.0–99.8%)**	0	n/a
Delaney et al., 2011	Clearview Complete	Whole blood	384	**98.7% (97.0–99.6%)**	103	100% (97.1–100%)
		Plasma	383	**99.0% (98.3–99.8%)**	103	100% (97.1–100%)
	Clearview Stat-Pak	Plasma	383	**98.4% (97.4–99.7%)**	106	100% (97.1–100%)
	OraQuick Advance	Whole blood	386	**99.2% (97.8–99.8%)**	106	100% (97.1–100%)
		Oral fluid	386	**97.7% (95.6–98.8%)**	106	100% (97.1–100%)
		Plasma	258	**100% (98.9–100%)**	69	100% (95.8–100%)
	Multispot	Plasma	376	**100% (99.2–100%)**	103	100% (97.1–100%)
	Reveal G3	Serum	383	**100% (99.2–100%)**	103	100% (97.1–100%)
	Uni-Gold Recombigen	Plasma	384	**100% (99.2–100%)**	106	100% (97.2–100%)
Merchant et al., 2014^a^	Uni-Gold Recombigen	Not specified	98	**98.0% (92.8–99.8%)**	0	n/a
	OraQuick Advance	Not specified	98	**90.8% (83.2–95.7%)**	0	n/a
	Reveal G3	Not specified	98	**86.7% (78.3–92.7%)**	0	n/a
	Clearview Complete	Not specified	56	**85.7% (73.8–93.6%)**	0	n/a
	Clearview Stat-Pak	Not specified	53	**79.2% (65.9–89.2%)**	0	n/a
Fogel et al., 2017^b^	OraQuick Advance	Not specified	207	**95.1% (91.3–97.7%)**	0	n/a
	Uni-Gold Recombingen	Not specified	207	**95.1% (91.3–97.7%)**	0	n/a
Siedner, 2020 (current)	Abon	Whole blood	157	**96.6% (92.8–98.8%)**	214	91.6% (88.3–94.3%)
	Advanced Quality	Whole blood	157	**99.3% (95.2–99.7%)**	214	95.4% (92.8–97.3%)

a Merchant et al. repeatedly test individuals (*n*=27) over time. Reported sensitivity estimates do not account for clustering of repeated testing.

b Fogel et al. assessed trial participants who previously testing positive by RDTs. Both nonreactive (*n*=1) and weakly reactive (*n*=9) results were considered negative. Sensitivity of the assays with weak bands considered positive was 99.5% (95% CI 97.3–100%).

Our data, along with prior studies that directly compared RDT sensitivity by ART use, are less suggestive of a large decrease in sensitivity due to ART use (Table 4). For example, a prior study from the US comparing 6 RDTs to an enzyme immunoassay/Western blot sequential reference standard in 386 individuals found >98% sensitivity in all assays among those on ART (Delaney et al., 2011). Although none of the assays showed statistically significant differences in test sensitivity by ART status, second-generation tests had larger nominal reductions in sensitivity for those on ART than third-generation tests, for which sensitivity was 100% for all assays. Of note, that study did demonstrate poorer sensitivity among people on ART for RDTs using oral fluid as an analyte (97.7% versus 100%), which is known to be less sensitive than blood specimens (Pant Pai et al., 2012). Although the assays were routinely 100% sensitive among those not on ART (*n*=106), there was no statistically significant difference in assay sensitivity by use or nonuse of ART. In our study, we found that 9 (5%) of 157 individuals currently in HIV care and on ART based on clinic records tested negative on all 3 assays studied (immunoassay and 2 RDTs). We also found a moderately increased sensitivity of RDTs compared to an immunoassay in people currently on ART versus those never exposed to ART.

The overall sensitivity of our RDTs reported (93–96%) was lower than that reported in many community-based studies (Molesworth et al., 2010; Jackson et al., 2013) and below WHO recommendations for near-perfect sensitivity for RDTs (Consolidated guidelines on HIV testing services, 2015). Nonetheless, our results are similar to other field-based studies comparing RDTs to immunoassays in the region (Wolpaw et al., 2010; Kufa et al., 2017). In 1 field-based study, low test sensitivity was drastically improved after implementation of augmented quality control procedures and a repeat testing algorithm (Bock et al., 2017). Testing was done in our study by lay health workers at participant homes. Decreased test accuracy has also been reported previously in field settings owing to difficult interpretation of weak test bands, high rates of indeterminate results (i.e., absent control band), and lower test accuracy among lay workers and nurses compared to laboratory staff (Gray et al., 2007; Klarkowski et al., 2009; Kagulire et al., 2011; Mwangala et al., 2016). Divergent test results in field sites compared to clinical and laboratory settings deserve further attention and likely have implications for the emergence of home and self-based testing programs (Sabapathy et al., 2012; Pant Pai et al., 2013).

Our study has a number of limitations. We conducted HIV immunoassay testing on dried blood spots, which might decrease the sensitivity of our reference standard assay. If the sensitivity of the immunoassay was lower than would have been expected with samples from serum or plasma specimens, this might have affected our estimate of RDT specificity. Moreover, we conducted confirmatory testing with only a single immunoassay in contrast to WHO guidelines, which suggest use of 2 confirmatory assays (World Health Organization, 2019). The fourth-generation EIA we used detects both antibody and antigen, as well as both HIV 1 and 2. Although the test does not distinguish HIV-1 from HIV-2, it is known that HIV-1 subtype C is dominant in South Africa, and a previous study found no evidence of HIV-2 in KwaZulu Natal (Singh et al., 2013).

RDTs were performed by lay health workers at home and might not reflect the diagnostic validity of these tests in a laboratory or clinical research setting. Our study also should be interpreted in the context of routine presentation and care of HIV and should not be generalized to acute treatment or cure studies, which often involve individuals without adequate humoral responses to detect HIV infection using serologic assays (Henrich et al., 2013; Fransen et al., 2017). Finally, we used clinical records to assess ART use, which are an imperfect measure of recent ART use.

In summary, we detected a similar sensitivity of RDTs in individuals taking ART compared to treatment-naïve individuals in a programmatic setting in rural South Africa. Our results support continued use of RDTs for population-based studies of HIV epidemiology, as well as for individuals who are transferring care or reinitiating therapy. Further attention should be paid to the test performance of RDTs in field-based settings to ensure adequate performance of the most common modality of HIV testing in such settings.

The following are the supplementary data related to this article.

Supplementary Table 1 Results of enzyme immunoassay and rapid diagnostic HIV tests overall and by antiretroviral therapy use status. Supplementary Table 1
