# Supplementary material for: Does antiretroviral therapy use affect the accuracy of HIV rapid diagnostic assays? Experience from a demographic health and surveillance site in rural South Africa
Source: Diagn Microbiol Infect Dis. 2020 Jun;97(2):115031. doi: 10.1016/j.diagmicrobio.2020.115031 (PMC7262582; doi:10.1016/j.diagmicrobio.2020.115031)
Supplement: Supplementary Table 1 — Results of enzyme immunoassay and rapid diagnostic HIV tests overall and by antiretroviral therapy use status. [file mmc1.docx]

**Supplemental Table 1.** Results of Enzyme immunoassay and rapid diagnostic HIV tests overall and by antiretroviral therapy use status.

|  | **All participants** | | | **Never on ART**^1^ | | | **Any Current or Prior ART**^2^ | | | **On ART >12 months**^3^ | | |
| --- | --- | --- | --- | --- | --- | --- | --- | --- | --- | --- | --- | --- |
| **Test strategy and result** | **Number of samples (%)** | **DBS ELISA result** | | **Number of samples (%)** | **DBS ELISA result** | | **Number of samples (%)** | **DBS ELISA result** | | **Number of samples (%)** | **DBS ELISA result** | |
|  |  | **Positive** | **Negative** |  | **Positive** | **Negative** |  | **Positive** | **Negative** |  | **Positive** | **Negative** |
| **All Participants** | 6802 (100%) | 550 | 6252 | 6613 | 371 | 6242 | 189 | 179 | 10 | 157 | 148 | 9 |
| Abon RDT |  |  |  |  |  |  |  |  |  |  |  |  |
| Negative | 6275 (92.3%) | 37 | 6238 | 6259 (94.7%) | 31 | 6228 | 16 (8.5 %) | 6 | 10 | 14 (8.9 %) | 5 | 9 |
| Positive | 521 (7.7 %) | 513 | 8 | 348 (5.3 %) | 340 | 8 | 173 (91.5%) | 173 | 0 | 143 (91.1%) | 143 | 0 |
| **Total** | 6796 (100%) | 550 | 6246 | 6607 (100%) | 371 | 6236 | 189 (100%) | 179 | 10 | 157 (100%) | 148 | 9 |
| Advanced Quality RDT |  |  |  |  |  |  |  |  |  |  |  |  |
| Negative | 6265 (92.1%) | 20 | 6245 | 6252 (94.6%) | 17 | 6235 | 13 (6.9 %) | 3 | 10 | 10 (6.4 %) | 1 | 9 |
| Positive | 535 (7.9 %) | 530 | 5 | 359 (5.4 %) | 354 | 5 | 176 (93.1%) | 176 | 0 | 147 (93.6%) | 147 | 0 |
| **Total** | 6800 (100%) | 550 | 6250 | 6611 (100%) | 371 | 6240 | 189 (100%) | 179 | 10 | 157 (100%) | 148 | 9 |
| Parallel RDT |  |  |  |  |  |  |  |  |  |  |  |  |
| Negative | 6252 (92.0%) | 16 | 6236 | 6240 (94.5%) | 14 | 6226 | 12 (6.3 %) | 2 | 10 | 10 (6.4 %) | 1 | 9 |
| Positive | 513 (7.6 %) | 509 | 4 | 341 (5.2 %) | 337 | 4 | 172 (91.0%) | 172 | 0 | 143 (91.1%) | 143 | 0 |
| Discordant | 29 (0.4 %) | 25 | 4 | 24 (0.4 %) | 20 | 4 | 5 (2.6 %) | 5 | 0 | 4 (2.5 %) | 4 | 0 |
| **Total** | 6794 (100%) | 550 | 6244 | 6605 (100%) | 371 | 6234 | 189 (100%) | 179 | 10 | 157 (100%) | 148 | 9 |

^1^Individuals who have no record in TIER. ^2^Individuals who have a record in TIER of ever being on ART. ^3^Individuals who have a record in TIER of an ART visit <6 months before the rapid test, and have been on ART for >1 year.
